# Establishment of a Cre-rat resource for creating conditional and physiological relevant models of human diseases

**DOI:** 10.1007/s11248-020-00226-7

**Published:** 2021-01-22

**Authors:** Huimin Zhang, Qi Zheng, Ruby Yanru Chen-Tsai

**Affiliations:** Applied StemCell, Inc., Milpitas, CA USA

**Keywords:** Transgenic rat, Cre, CRISPR/Cas9, TARGATT

## Abstract

The goal of this study is to establish a Cre/loxP rat resource for conditional and physiologically predictive rat models of human diseases. The laboratory rat (*R*. *norvegicus*) is a central experimental animal in several fields of biomedical research, such as cardiovascular diseases, aging, infectious diseases, autoimmunity, cancer models, transplantation biology, inflammation, cancer risk assessment, industrial toxicology, pharmacology, behavioral and addiction studies, and neurobiology. Up till recently, the ability of creating genetically modified rats has been limited compared to that in the mouse mainly due to lack of genetic manipulation tools and technologies in the rat. Recent advances in nucleases, such as CRISPR/Cas9 (clustered regularly-interspaced short palindromic repeats/CRISPR associated protein 9), as well as TARGATT™ integrase system enables fast, efficient and site-specific introduction of exogenous genetic elements into the rat genome. Here, we report the generation of a collection of tissue-specific, inducible transgenic Cre rats as tool models using TARGATT™, CRISPR/Cas9 and random transgenic approach. More specifically, we generated Cre driver rat models that allow controlled gene expression or knockout (conditional models) both temporally and spatially through the Cre-ERT2/loxP system. A total of 10 Cre rat lines and one Cre reporter/test line were generated, including eight (8) Cre lines for neural specific and two (2) lines for cardiovascular specific Cre expression. All of these lines have been deposited with the Rat Resource and Research Center and provide a much-needed resource for the bio-medical community who employ rat models for their studies of human diseases.

## Introduction

Transgenic animals have long been used in medical research as tools to demonstrate the underlying pathological or genetic processes involved in many human diseases. The benefit gained from studying animal models has proved invaluable to many areas of medicine, from models of cardiovascular diseases (Bader 2010), neural degenerative diseases (Flood et al. 2007) to depression (Overstreet 1993), and more recently the potential of cell therapy to restore function to the body following spinal cord damage (Rasiman and Li 2007; Chen et al. 2001).

Various forms of transgenic animals can be made to delete an existing gene (knockout models), or to introduce a new gene, or have a gene expressed at higher than normal levels (knockin models), and to have a gene expressed or knocked-out in a specific-tissue and/or at a specific time (conditional transgenic models). These conditional transgenic models offer a more accurate representation of the biology of a living organism. This is because cellular diversity in a developing organism is achieved by spatial and temporal regulation of gene expression determined by genetic programs and cellular signaling. As a result, the overall function of genes is often complex, governed by precise spatial and temporal activity, which are difficult to dissect without proper genetic tool models providing similar complexity of control of expression, disruption or overexpression of a gene of interest (Lewandoski 2001; Lobe and Nagy 1998). Uncontrolled transgenic expression of a given gene in all tissues or even in restricted cell types may cause embryonic lethality that prohibits further studies at later stages of development or in adults, especially for studies of neural cognitive and behavioral mechanisms which require adult or aged animals. In mice, over 30% of uncontrolled, straight gene knockout models are embryonic lethal or sub-viable (data from mouse model consortium KOMP and IMPC).

Tissue/cell-specific gene knockout or gene expression can be achieved by a two-step process. The first step is to introduce loxP sites flanking a functionally essential genomic sequence followed by a cell type-specific Cre recombinase-mediated excision of the loxP-flanked (“floxed”) sequence (Sauer 1987; Sauer and Henderson 1988). The same strategy can be used for cell type-specific overexpression of a transgene, when a strong, ubiquitous promoter is separated from the coding region of a gene by a loxP-flanked ‘STOP’ cassette (Lakso et al. 1992; Orban et al. 1992). As a result, Cre/loxP mediated recombination provides spatial-controlled gene expression, which in most cases, is irreversible once Cre expression has switched on and recombination has occurred.

The Cre-ER system allows conditional gene expression that combines tissue/cell-specific gene regulation with inducible system, where Cre “switch-on” is independently controlled from lineage specificity (Feil et al. 1996, 1997). This system takes advantage of the nuclear localization capability of the estrogen receptor ligand-binding domain. The Cre recombinase is fused to a mutant ligand-binding domain (named CreER), which lost its ability to bind endogenous estrogen, but instead binds tamoxifen (an estrogen antagonist). In the presence of tamoxifen, the Cre fusion protein translocates into the nucleus where it recombines loxP sites and deletes the floxed chromosomal sequence. The properties of CreER were further improved to decrease the background activity in the absence of inducer and to increase the sensitivity and efficiency of tamoxifen-induced recombination. The CreERT2 recombinase, which contains the human estrogen receptor ligand-binding domain with a G400V/M543A/L544A triple mutation, is currently the best version recommended for inducible models (Feil et al. 2009).

In the last several decades, tremendous efforts were put into generating Cre-driver mice all over the world. Over 100 Cre driver mice can be purchased from the Jackson Laboratory (http://jaxmice.jax.org). In comparison very few Cre rats are available due to technical difficulties. Recent advances in nuclease-based genome editing methods including Zinc-Finger Nuclease (ZFNs), Transcription Activator-Like Effector Nuclease (TALENs), and particularly CRISPR/Cas9 (Clustered Regularly Interspaced Short Palindromic Repeats and CRISPR-Associated protein 9) have revolutionized the way genetic animal models are generated (Meyer et al. 2010, 2012; Cong et al. 2013; Jinek et al. 2013; Mali et al. 2013). For example, CRISPR/Cas9 microinjected into zygotes works effectively for inserting small nucleotide sequences, such as the 34 bp loxP site, directly into the genome thereby bypassing the requirement for homologous recombination steps in rat ES cells. Rat models containing a floxed allele can be generated in as little as in 6 months.

The other component for conditional rat models is a Cre-driver rat. Although such Cre rat can be generated by random transgene integration via pronuclear injection, random insertion subjects the transgene to position effect. Because of that, multiple lines need to be generated and characterized, sometimes for many generations, in order to obtain a usable line with good, stable gene expression. Some of these problems can be overcome by using artificial chromosomes (such as bacterial, yeast), since they allow a much greater amount of genetic information to be incorporated and can include a “buffer” region (West et al. 2002) around the gene of interest to protect it from position effect. But this method is far less efficient. Several publications described generation of Cre driver rats using the BAC strategy (Witten et al. 2011; Weber et al. 2011). They are tyrosine hydroxylase (TH)-Cre, choline acetyltransferase (Chat)-Cre, and serotonergic Tph2-Cre. More recently Brown et al. (2013) reported conditional gene knockout rat models using ZFN technology. In brief, the authors generated floxed-alleles at three targets including Grin1, crhr1 and Tp53. They also generated a Cre-driver rat by knocking-in Cre under the endogenous TH (tyrosine hydroxylase) promoter. When floxed Grin1 rats were bred with the Th-Cre driver rats, Cre-dependent excision of Grin1 from the genomic DNA of the brain and adrenal gland was detected, coinciding with TH promoter tissue-specific activity. Using a similar strategy, the company Horizon Discovery has so far generated 7 Cre-driver rats including TH-Cre, DAT-Cre, CamlIa-Cre, Vgat-Cre, Tph2-Cre, VIP-Cre, and 5Ht3a-Cre rats.

Although ZFNs has been shown to work in generating these Cre driver rats, their application is limited mainly due to the relatively high cost and the requirement of expertise in designing and validation of the ZFNs. CRISPR, on the other hand, is much easier to use and more cost effective, but is most efficient at inserting small fragments such as those supplied as single stranded oligonucleotide (ssODN) of templates. For insertion of larger double stranded DNA, such as CreERT2 with a cell-specific promoter (usually > 6 kb), CRISPR/HDR is not efficient at its current state (Singh et al. 2015).

To address these limitations, we developed an improved integrase-based technology, trademarked as “TARGATT™” that allows fast generation of site-specific knockin rat models with lower cost. The phiC31 and Bxb1 integrase from the *Streptomyces* phage and *Mycobacterial* phage, respectively, catalyze recombination between two non-identical sites, *attB* and *attP* (Groth and Calos 2004; Keravala et al. 2006). This feature, along with the lack of a corresponding excisionase enzyme, makes the recombination reaction unidirectional, ensuring that constructs integrated into the genome do not act as substrates for the reverse reaction. The result is an improvement in integration efficiency compared with random integration or other recombinase system such as Cre/loxP, which works for both DNA deletion and insertion. Using TARGATT™ in mice we have successfully generated site-specific transgenic knockin models for over 100 DNA constructs with up to 40% and an average of ~ 10% insertion efficiency (Tasic et al. 2011; Fan et al. 2012; Guenther et al. 2014).

In this study, we use both CRISPR and TARGATT™ technology and generated a total 10 tissue-specific, inducible Cre-rat lines and a LoxP-stop-loxP test line. These lines are available from the RRRC to benefit researchers who use rat models to advance their studies.

## Materials and methods

### Plasmid and constructs

For gene knockin using TARGATT, insertion plasmids (except for the reporter/test line #21) contain an attB site for site-specific recombination with attP docking site at the TARGATT rat genome, an expression cassette consisting of a lineage-specific promoter, a nuclear CreERT2 cDNA, a reporter mCherry, and a generic intron-polyA. All rat lines contain the same CreERT2-mCherry cassette except for the lineage-specific promoter/enhancer sequences.

Construct for the reporter line #21 is in a configuration of “attB-pCA-loxP-Stop-loxP-GFP-2A-lacZ” (Fig. 6). The pCA promoter (CMV-Beta-actin promoter) is separated from the reporter genes GFP and LacZ by a “Stop” cassette flanked by loxP sites. The “stop cassette” is 1.3 kb in size composed of the 550 bp C-terminal sequence of yeast His3 gene, 825 bp of the SV40 polyadenylation signal region, and a synthetic oligonucleotide (5′-GATCTGACAATGGTAAGTAAGCTT-3′, where ATG is a false translation initiation signal and GTAAGT is a 5′splice donor site) (Lakso et al. 1992).

For gene knockin using CRISPR/Cas9, two plasmids were constructed; one contains gRNA that targets the specific genomic locus and the other is donor plasmid containing CreERT2-mCherry cassette. The Cre cassette (CreERT2-mCherry) is 2.8 kb in size and single-strand DNA is gel-purified and used for microinjection.

Table 1 lists all the validated gRNAs for each of the CRISPR donor constructs. Validation was done in rat embryos by microinjection of the gRNA with Cas9 protein. Indel activity was calculated by percentage of embryos containing indels. At first two gRNAs were tested per donor construct with gRNA activity required to be > 30% to pass QC. If neither of the two gRNAs showed > 30% activity, new gRNAs were designed and tested again until a qualified gRNA was identified. All CRISPR donor constructs contain the same CreERT2-mCherry cassette except for the flanking homologous arms. Both 5′ and 3′ arms are about 100 bp, homologous to the corresponding genomic sequence where the insertion takes place. The CreERT2-mCherry cassette is designed to insert at the 3′ end of the tissue-specific gene connected by P2A sequence, therefore co-expressing with the tissue-specific gene.

**Table 1:** gRNA validation data

Line/gene	gRNA	Sequence	Indel %
#2 Wnt1	g3	GTACTGCACGAGTGTCTATG	100
#8 Pomc	g3	GCGCACAAGAAGGGCCAGTG	57
#9 Hb9	g2	TGCCCCAGTAGTTGTCCCAA	100
#10 Drd1a	g2	ATGAGGACCCAATATTCAAG	100
#12 Gad67	g2	ATTACGGTTCTGCAAAGGGG	100
#19 Tie2	g2	TCTGCTGAAGAAGCAGCCTA	100

### Animals

Animals used in this study were maintained at the animal facility at NASA Ames Research Center (Mountain View, CA, USA). All protocols involving use of animals were approved by the Institutional Animal Care and Use Committee (IACUC) of the NASA Ames Research Center. Sprague Dawley rats were purchased from Charles River (strain code 001) (Wilmington, MA). Nestin-Cre mice were purchased from Jackson Lab (stock # 003771-B6.Cg-Tg(Nes-cre)1Kln/J).

### Generation of rat models by microinjection

To generate knockin rats, we performed cytoplasmic microinjection of the CRISPR/Cas9 constructs into rat one-cell embryos. CRISPR microinjection mixture contained 50 ng/μl gRNA, 200 ng/μl donor ssODN, and 20 ng/μl microinjection validated Cas9 protein. TARGATT microinjection mixture contained 50 ng/μl integrase mRNA, 5 ng/μl donor plasmid in RNase-free microinjection TE buffer. After microinjection, embryos were cultured in KSOM medium for a minimum of half an hour and then implanted into the oviduct of pseudo pregnant female rats.

Genotyping of live born pups were performed by PCR using specific primer sets, usually 3 sets, each detecting 3′ junction, 5′ junction, and internal sequence.

### Immunohistochemistry

To characterize Cre expression, brain tissues were harvested, embedded and sectioned according to standard histology protocols. Sections were stained with anti-Cre polyclonal antibody (ThermoFisher Cat# PA5-32244) and goat anti-rabbit IgG 2nd antibody conjugated with Alexa fluor 594 (ThermoFisher Cat#A-11037), and then were imaged using fluorescence microscope.

## Results

### Generation of docking site ready (DSR) rat strain, or TARGATT™ rat strain

**Fig. 1:**
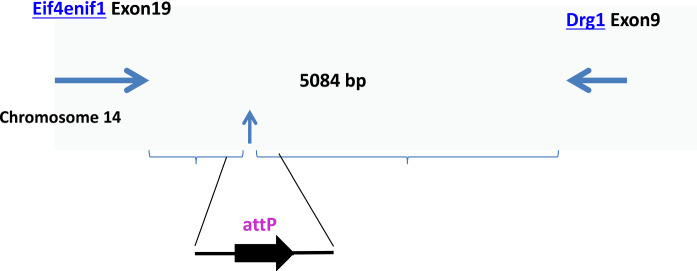
Rat chromosomal locus *rH11 (rChr14-Eif).* Insertion site is 700 bp away from 3′ UTR of *Eif4enif1* and 4.5 kb away from *Drg1*

In order to use the TARGATT™ technology, the first step was to place an *attP* recognition sequence for phiC31 integrase into a genomic safe harbor locus in rat genome. Based on our published studies of the mouse “hot spot” *H11* locus (Tasic et al. 2011, 2012; Fan et al. 2012; Guenther et al. 2014) and its human orthologous *hH11* locus (Zhu et al. 2013) as well as its pig orthologous *pH11* locus (Ruan et al. 2015), the gene arrangement and molecular structure of this genomic locus is highly conserved among mouse, pig, and human. We predicted that the rat orthologous locus is likely to support high-efficiency gene integration and gene expression, similar to the mouse, pig, and human locus. Using the rat and mouse sequences and their surrounding genes, we identified that the rat orthologous locus is on rat chromosome 14, and is named as *rH11*. Figure 1 shows the genomic structure of the *rH11* locus. The *H11* locus is in an intergenic region and between two highly expressed genes. The mouse *H11* locus has been well characterized to support ubiquitous, tissue-specific or inducible gene expression depending on the promoter used.

**Fig. 2:**
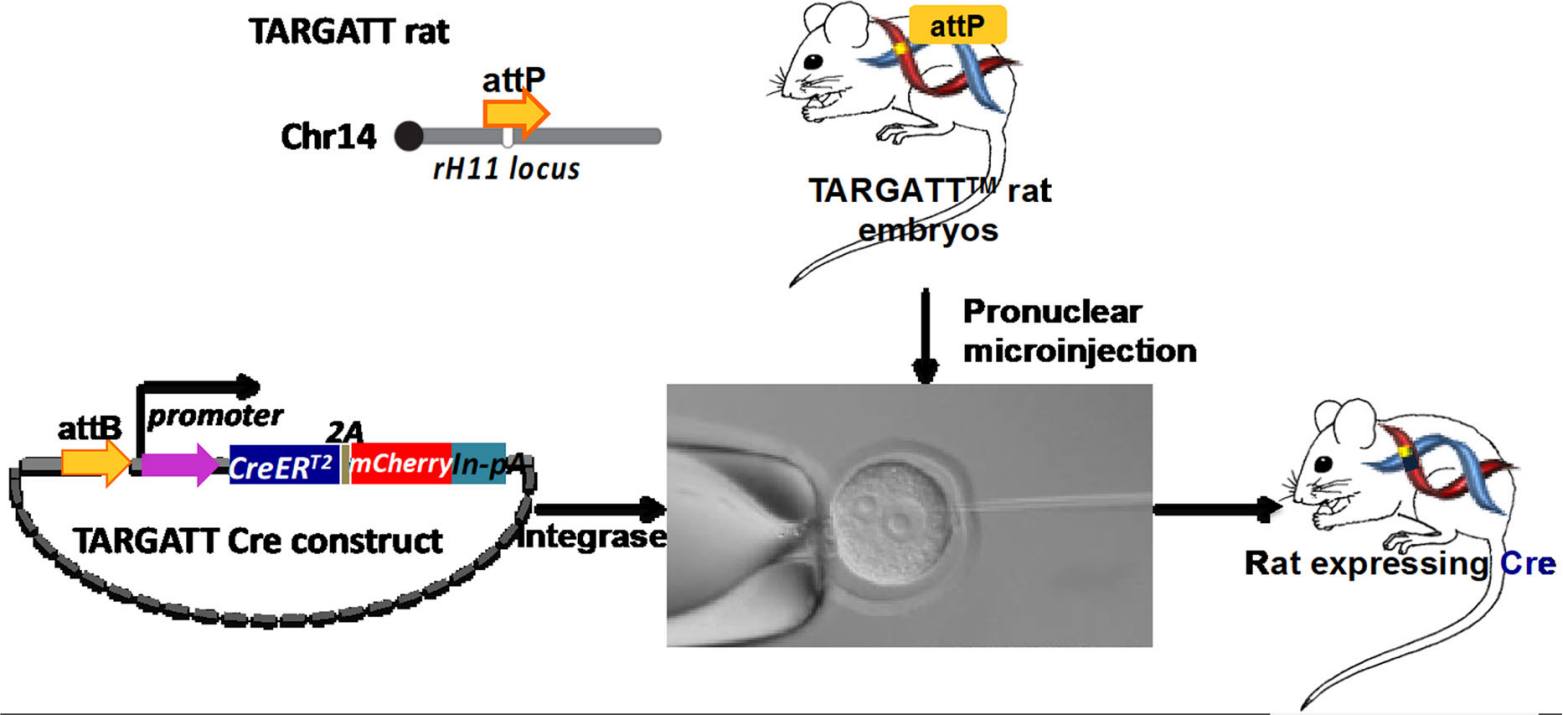
Scheme on generating tissue-specific Cre driver lines. TARGATT Cre construct contains tissue-specific promoter driving nuclear/inducible CreERT2/mCherry with a SV40 intron-polyA cassette (In-pA), and attB site. TARGATT rat embryos will be microinjected with a mixture of TARGATT Cre DNA and integrase mRNA or protein. Live born animals will be screened for the presence of the Cre expression cassette

The “docking site ready (DSR)” rat strain, or TARGATT™ rat strain was generated using CRISPR/Cas9. With integrase, these TARGATT™/DSR rats are used as embryo donors to site-specifically integrate any gene of interest on an *attB*-containing vector via pronuclear injection (Fig. 2).

### Generation of the Cre lines

#### Details of rat models

As rats are considered better models than mice in studies of neural behavioral, cognitive studies and in cardiovascular disease studies, we generated 8 neural lineage-specific and 2 cardiovascular lineage-specific Cre lines. We used inducible CreERT2 so that Cre activity is tightly controlled in space and time. The CreERT2 recombinases are inactive, but can be activated by the synthetic estrogen receptor ligand 4-hydroxytamoxifen (OHT), allowing for external temporal control of Cre activity. Indeed, by combining tissue-specific expression of a CreERT2 recombinase with its tamoxifen-dependent activity, the excision of floxed chromosomal DNA can be controlled both spatially and temporally by treating the rat with tamoxifen, which is metabolized to OHT. We also included a fluorescent reporter mCherry (Shaner et al. 2005) in the Cre cassette linked by a T2A sequence (CreERT2-2A-mCherry) so that Cre and mCherry are co-expressed in the same cells. The “self-cleaving” T2A sequence mediates ribosome-skipping events, enabling the generation of two separate peptide products CreERT2 and mCherry form one mRNA (Ryan et al. 1991; Lange et al. 2012; Minskaia and Ryan 2013).

We divided the 11 constructs into two groups based on the size of the DNA fragment insertion and whether or not the promoter is well-defined. Group (1) includes constructs with well-defined tissue-specific promoters; and Group (2) includes constructs with less well-defined tissue-specific promoters. The details of the constructs, rat models and status are listed in Table 2. For Group (1), we used TARGATT site-specific gene insertion technology to generate the models, whereas for Group (2), we used CRISPR method to insert the CreERT2-mCherry cassette at the tissue-specific gene locus. This parallel approach enabled us to generate the models in the most efficient and timely fashion.

**Table 2:** Rat model list

Group 1 TARGATT			Group 2 CRISPR/Cas9	
Rat line (list#) status	Tissue/cell-specificity	Promoter (reference)	Rat line (list#) Status	Tissue/cell-specificity (reference)
PDGF-CreERT2 (#5) Founders/F1(+mCherry) (random insertion)	Neurons of cortex, cerebellum, brain stem, spinal cord and olfactory bulb	1.425-kb of the human PDGF B-chain gene (Sasahara et al. 1991)	Wnt1-CreERT2 (#2) Founders**/**F1s	Developing neural crest and midbrain (Chou et al. 2013)
MOR23-CreERT2 (#7) Founders/F1 (+mCherry)	Olfactory sensory neuronal lineage	2.2-kb of mouse MOR23 promoter (Li et al. 2004; Vassalli et al. 2002)	Pomc-CreERT2 (#8) Founders/F1(+mCherry)	Neurons of arcuate nucleus (hypothalamus) and solitary tract nucleus (hindbrain) (Young et al. 1998)
GFAP-CreERT2 (#16) Founders/F1(+mCherry)	Astrocytes in CNS	2.2-kb human GFAP (glial fibrillary acidic protein) (Brenner et al. 1994)	HB9-CreERT2 (#9) Founders/F1(+mCherry**)**	Motor neurons (Arber et al. 1999; Yang et al. 2001; Nakano et al. 2005; Tasic et al. 2011)
SMHC-CreERT2 (20) Founders/F1(+mCherry)	Vascular smooth muscle cells	2.3-kb rabbit smooth muscle myosin heavy chain promoter (Franz et al. 1999)	Drd1a-CreERT2 (#10) Founders/F1	Dopamine D1 receptor-expressing neurons (Zhang et al. 2006)
CA-Lox-STOP-Lox-GFP-LacZ (21) Founders/F1 (random)	Cre reporter/test line expressing GFP and lacZ	1.7-kb CA (CMV-beta-actin) promoter (Tasic et al. 2011)	GAD67-CreERT2 (#12) Founders/F1	GABAergic neurons, islet cells and spermatocytes (Kobayashi et al. 2003; Rasmussen et al. 2007)
			Tie2-CreERT2 (#19) Founders/F1 (+mCherry)	Vascular endothelial cells (Schlaeger et al. 1997; Ohtsuki et al. 2005)

#### Lines made using TARGATT™

The four Cre lines generated using TARGATT are PDGF-CreERT2-mCherry (**#**5), MOR23-CreERT2-mCherry (**#**7), GFAP-CreERT2-mCherry (**#**16), and SMHC-CreERT2-mCherry (**#**20). Integration vectors all contain a tissue/cell-specific promoter driving CreERT2-mCherry with an intron and polyA cassette.

Line#5 (PDGF-CreERT2) contains the human PDGF beta-chain promoter to drive Cre expression in rats. This human promoter region was shown to drive reporter gene expression (CAT: Chloramphenicol acetyltransferase) mainly in the brain including cortex, cerebellum, brain stem, spinal cord and olfactory bulb, overlapping with antibody expression studies in the non-human primate brain (Sasahara et al. 1991).

Line#7 (MOR23-CreERT2) contains 2.2-kb of mouse MOR23 promoter (Li et al. 2004; Vassalli et al. 2002), which was shown to drive expression in olfactory sensory neurons in transgenic mice. Comparison of mouse and human MOR23 identified a number of conserved motifs upstream of transcriptional start site that direct expression specifically in the olfactory bulb in transgenic mice (Vassalli et al. 2002).

Line#16 (GFAP-CreERT2) contains a 2.2-kb 5′ flanking sequence derived from the human GFAP gene. This promoter was shown to direct reporter lacZ expression to astrocytes in the CNS in transgenic mice, as well as direct astrocyte-specific transcription in cultured human cells (Brenner et al. 1994). Given these data, the same promoter will most likely recapitulate the brain expression specificity in the proposed Cre rats.

Line#20 (SMHC-CreERT2) contains a 2.3-kb promoter of the rabbit smooth muscle myosin heavy chain (SMHC) gene. This promoter was shown to direct luciferase expression specifically in vascular smooth muscle cells of large arteries including coronary arteries in transgenic mice and rabbits (Franz et al. 1999). This promoter is likely to have a similar cell-specific activity in transgenic rats.

**Fig. 3:**
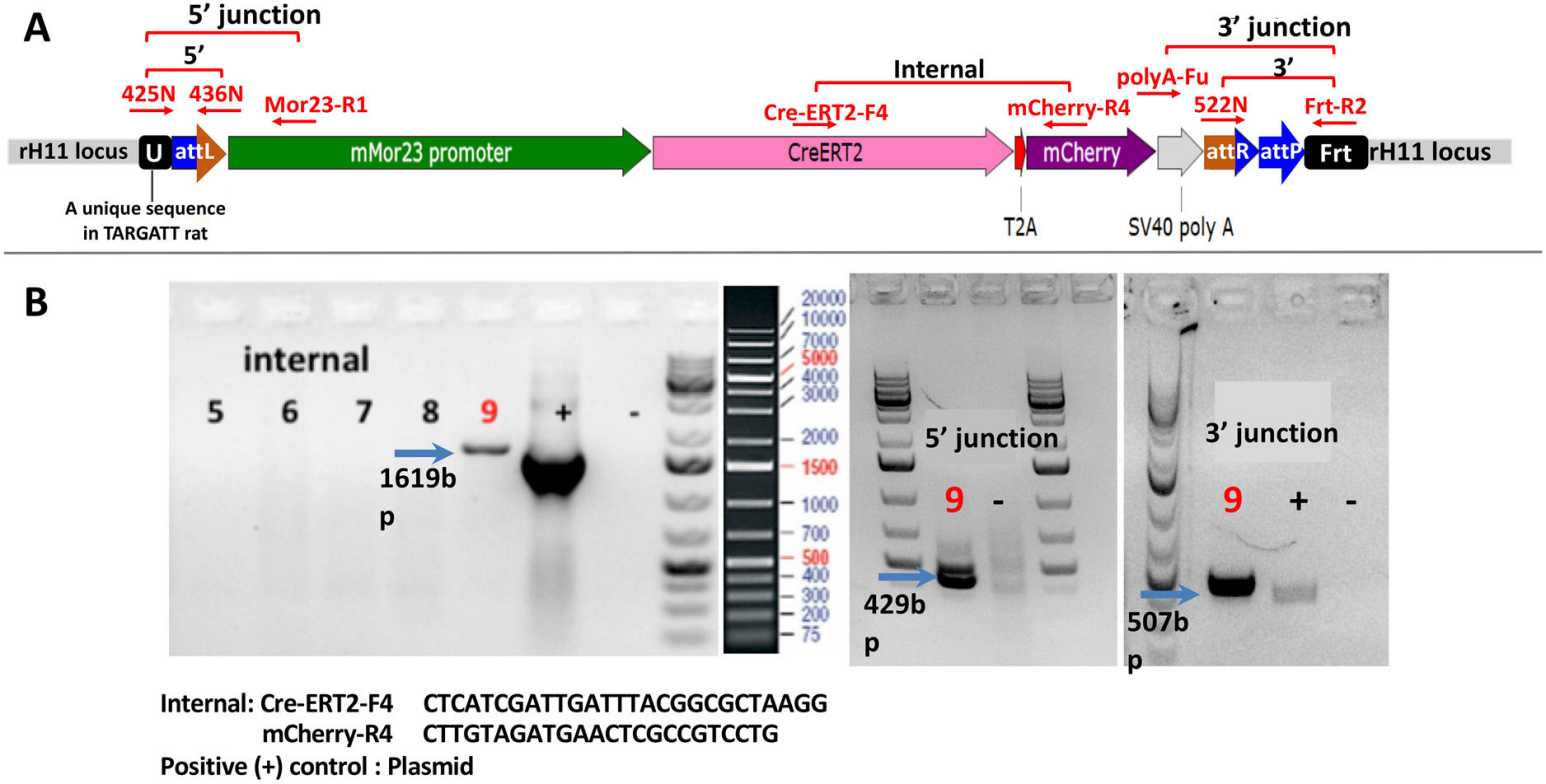
Scheme on generating Mor23-CreERT2 transgenic rat. **a** Genomic structure after TARGATT gene insertion at the attP site (blue). **b** Genotyping results using PCR primers (in red) amplifying internal, 5′ and 3′ junction of the transgene insertion. Fragments with correct size are indicated in blue arrows

Figure 3 shows the strategy and genotyping result of Mor23-CreERT2 model as an example of TARGATT approach. The integration vector was inserted at the *attP* site of the TARGATT rat via integrase mediated recombination between *attB* (on integration vector) and *attP* site (at genomic *rH11* locus) (Fig. 1). PCR using 3 primer sets followed by sequencing confirmed correct fragment size and sequence for internal, 5′ and 3′ junction of the transgene, indicating that #9 is a positive animal containing Mor23-CreERT2-mCherry at the safe genomic locus*, rH11*.

In general, TARGATT generated lines contain intact transgene insertion, but with some founders containing both site-specific and random insertion. In the case of line#5 (PDGF-CreERT2), only random insertion went germline.

#### Lines made using CRISPR

We generated six (6) Cre lines using CRISPR approach. They are Wnt1-CreERT2 (#2), Pomc-CreERT2 (#8), HB9-CreERT2 (#9), Drd1a-CreERT2 (#10), GAD67-CreERT2 (#12), and Tie2-CreERT2 (#19).

**Fig. 4:**
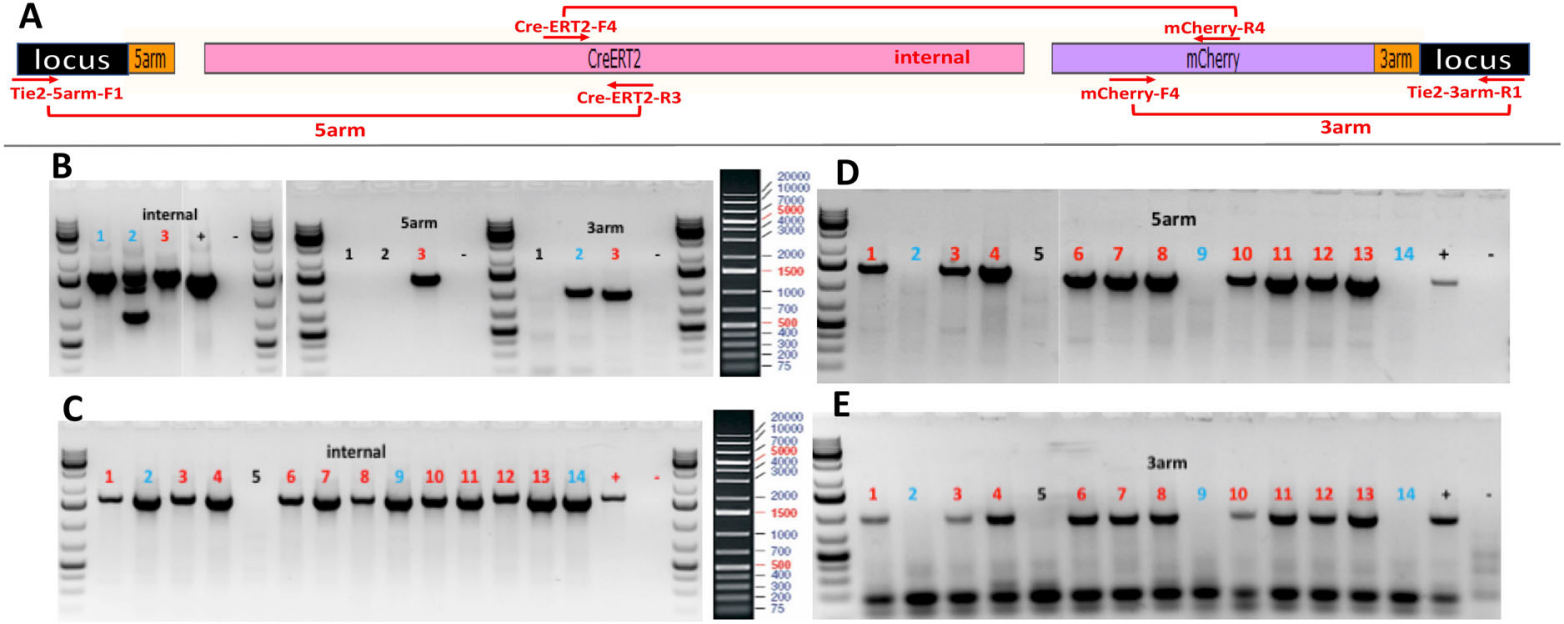
Scheme on generating Tie2-CreERT2 transgenic rat. **a** Genomic structure after CreERT2-mCherry insertion at the Tie2 locus using CRIPSR/Cas9 approach. Genotyping primers are labeled in red. **b** Genotyping results of potential founders using PCR primers amplifying internal, 5′ junction and 3′ junction for transgene insertion. #3 animal is confirmed to be a positive transgenic founder. **c**–**e** Genotyping results of F1 progeny of the # 3 founder using PCR primers amplifying internal (**c**), 5′ junction (**d**), and 3′ junction (**e**) for correct transgene insertion

Figure 4 shows the process, genomic map, targeting strategy and genotyping result of Tie2-CreERT2 model as an example of this CRISPR/Cas9 approach. The CreERT2-mCherry cassette is integrated at the gRNA cutting site before the stop codon of Tie2. PCR using 3 primer sets followed by sequencing confirmed correct fragment size and sequence for internal CreERT2-mCherry, 5′, and 3′ junction fragments, indicating that #3 is a positive founder. Further breeding generated multiple F1 positive animals (Fig. 4c–e).

**Fig. 5:**
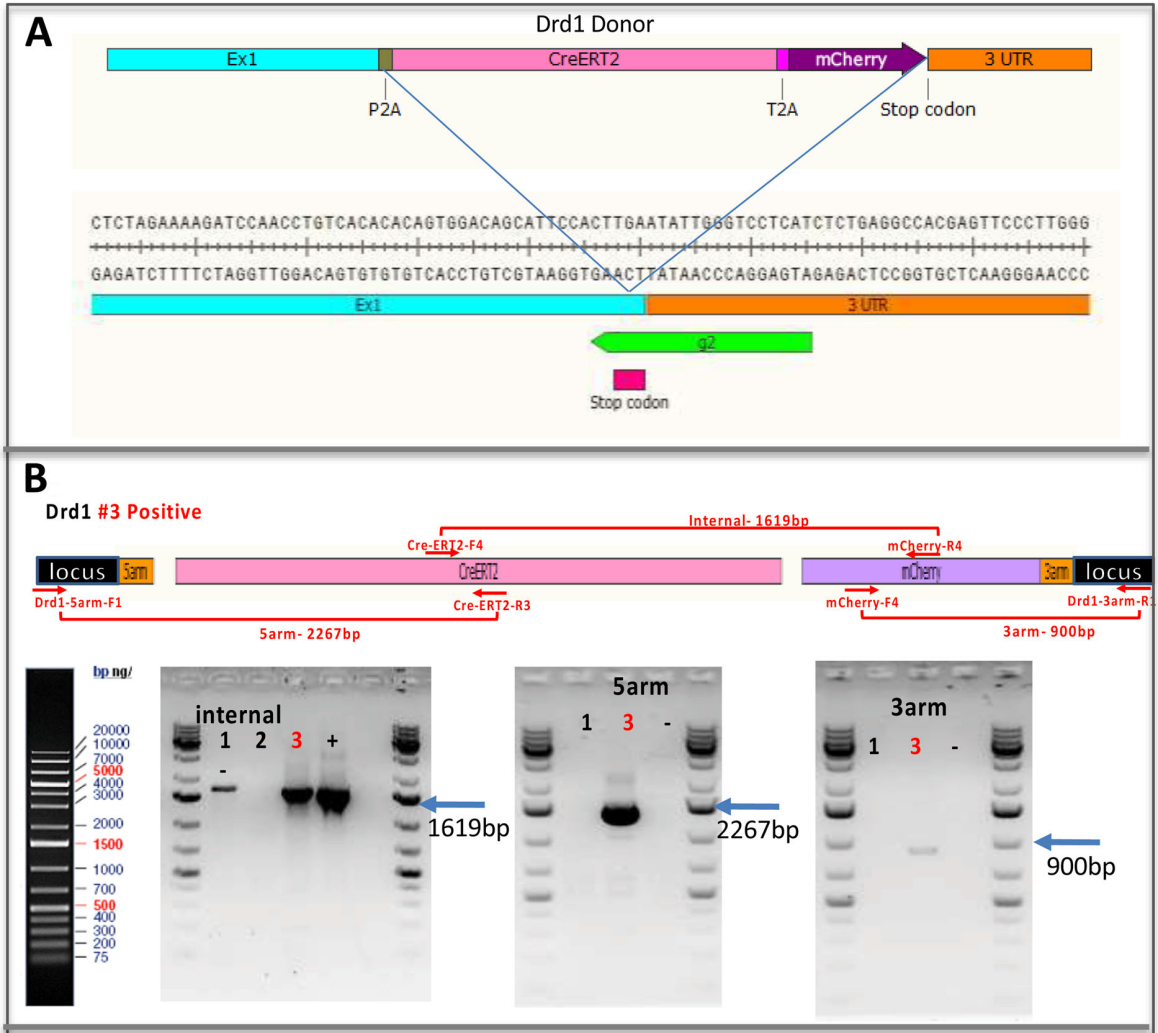
Scheme on generating Drd1a-CreERT2 transgenic rat. **a** Donor construct and gRNA targeting location. **b** PCR primers and genotyping results

Figure 5 shows the process, genomic map, targeting strategy and genotyping result of Drd1a-CreERT2 model as an example. The Drd1 donor is integrated at the gRNA cutting site which is right before the stop codon of Drd1. PCR using 3 primer sets followed by sequencing confirmed correct fragment size and sequence for internal CreERT2-mCherry, 5′ junction, and 3′ junction fragments.

For Drd1a-Cre and GAD67-Cre lines, we were able to detect internal transgene and 5′ junction fragment; but failed to amplify the 3′ junction fragment. We suspect that there may be indels or other mutations associated with CRISPR gene editing. Since mCherry is at the 3′ end of the transgene, its expression may get affected.

#### Cre reporter/test line (Line #21)

**Fig. 6:**
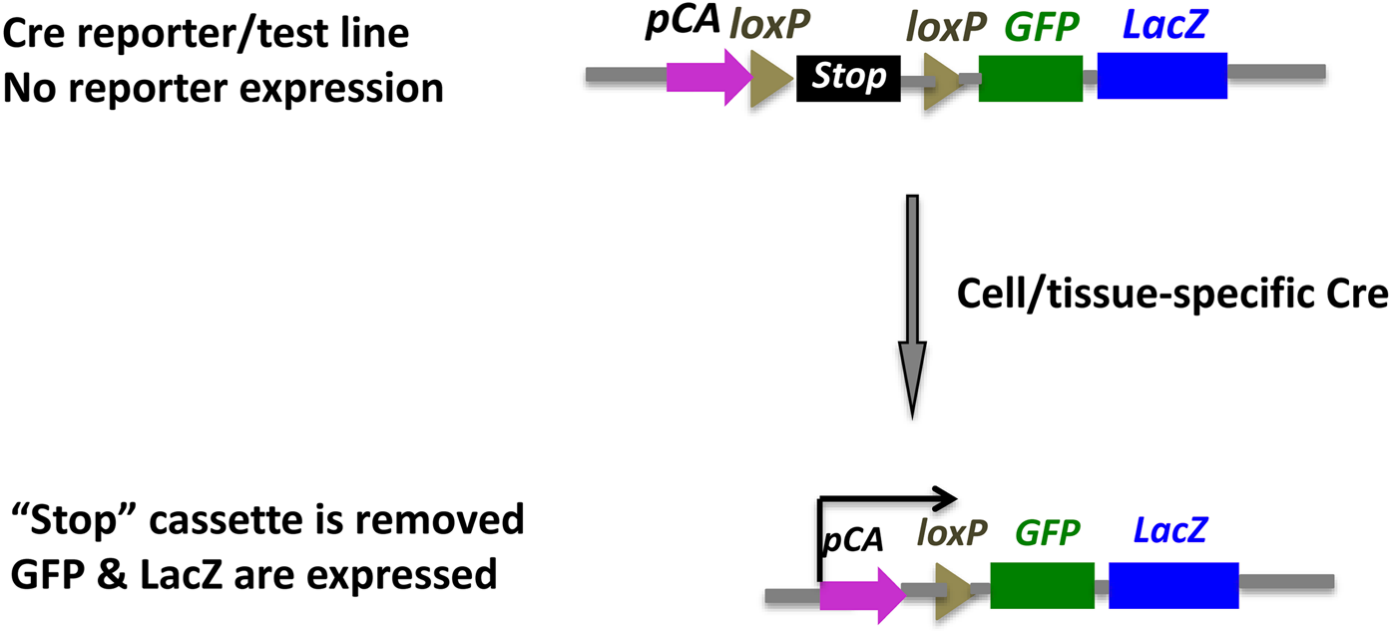
Cre reporter/test transgenic line. This line is used to test the function of Cre driver lines. The “LoxP-Stop-LoxP” cassette between the promoter pCA and GFP/LacZ reporters prevents expression of the reporters. Upon Cre recombination between the two loxP sites, the “stop” cassette is removed and GFP/LacZ reporters are expressed

This is a Cre reporter/test line that allows Cre recombinase functional testing. The transgene is in a configuration of “attB-pCA-loxP-Stop-loxP-GFP-2A-lacZ” (Fig. 6). GFP and LacZ are not expressed in the reporter line. When a Cre driver line is bred with the Cre reporter line, recombination of the two loxP sites causes excision of the “Stop” cassette, resulting in expression of GFP and LacZ reporters in the specific cells/tissues where Cre is expressed. The dual reporter system will allow both in *vivo* (GFP) and histological (LacZ staining) detection of gene expression.

Although this line was generated using the TARGATT approach with the intention of recombining the transgene into the *rH11* safe harbor locus, the transgenic founders and F1s analyzed contained only random insertion events.

### Transgenic efficiency using CRISPR and TARGATT

Table 3 is a summary of the microinjection experiments on the models generated either by CRISPR or TARGATT method. The lines generated by TARGATT have a low efficiency (~ 1%) compared to what was reported in TARGATT mice, which is up to 40% and averaged at 10% (Tasic et al. 2011). There are several possible causes of this unexpected outcome. The TARGATT knockin constructs in this study are between 8.3 and 8.9 kb range, which is relatively large in size. Large knockin usually has lower transgenic efficiency. The integrase mRNA used in the microinjection mix may not be in an optimal condition, affecting its integration activity. The fact that random transgenic founders were identified support the sub-optimal integrase activity assumption. The rat *H11* locus may have a lower accessibility to the TARGATT integrase enzyme compared to the mouse *H11* locus. Due to the scale of this project, we also stopped microinjection as soon as a site-specific transgenic founder was identified. Therefore, the dataset presented here may not accurately reflect the actual efficiency.

**Table 3:** Microinjection data

	Construct size (kb)	# Injected embryos	# Embryos transferred/#recipient	# Pups born	# Positive founders (%)	Note
*Targatt*						
PDGF-CreERT2 (#5)	8.3	107	81/3	31	1 (3%)	2 Random positives
MOR23-CreERT2 (#7)	8.8	153	110/3	15	1 (7%)	
GFAP-CreERT2 (#16)	8.7	77	43/2	24	1 (4%)	2 Random positives
SMHC-CreERT2 (20)	8.9	258	180/4	34	1 (3%)	1 Random positive
*CRISPR*						
Wnt1-CreERT2 (#2)	2.0	150	138/4	14	3 (21%)	No mCherry gene 1 founder has point mutation
Pomc-CreERT2 (#8)	2.8	778	620/14	85	3 (4%)	3 founders with deletions at junctions; 2 founders have point mutation
HB9-CreERT2 (#9)	2.8	135	101/2	10	1 (10%)	1 founder with point mutations; 1 founder has 40 bp deletion at 3’
Drd1a-CreERT2 (#10)	2.8	198	158/2	20	2 (10%)	
GAD67-CreERT2 (#12)	2.8	201	176/3	21	1 (5%)	1 founder has deletion in Cre; 1 founder has deletion at 3′ junction
Tie2-CreERT2 (#19)	2.8	106	83/2	9	1 (11%)	1 founder has point mutations

The lines generated using CRISPR method ranged from 4 to 21% in integration efficiency. This wide range may reflect that efficiency is locus dependent. We also observed point mutations in the transgene and/or indels/large deletions at the 5′ and/or 3′ junctions. The knockin ssDNA fragment size is 2.8 kb except for line #2 which is 2 kb.

### Characterization of the Cre driver lines and Cre reporter/test line

**Fig. 7:**
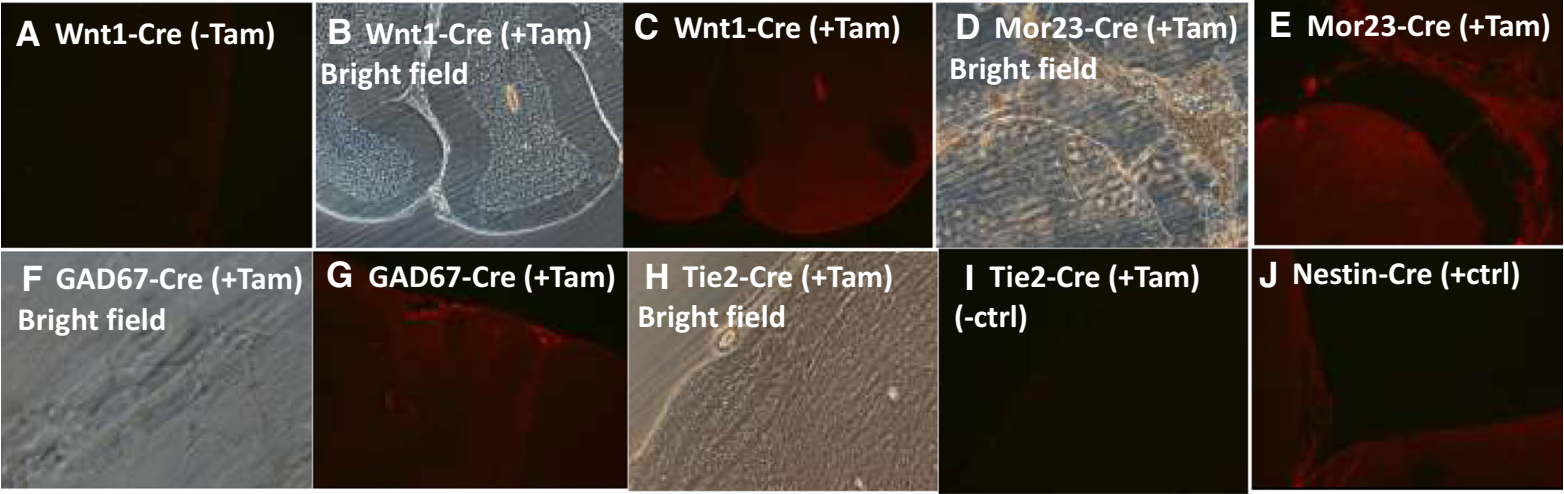
Cre expression in Wnt1-CreERT2, Mor23-CreERT2, GAD67-CreERT2, and Tie2-CreERT transgenic rat brain sections. Brain sections were stained with anti-Cre antibody and goat anti-rabbit IgG 2nd antibody conjugated with Alexa fluor 594. Cre was expressed in the brain sections in tamoxifen (+Tam) induced Wnt1-Cre (**c**), Mor23-Cre (**e**), and GAD67-Cre (**g**), but not in Tie2-Cre (**i**). Nestin-Cre transgenic mouse was used as a positive control for Cre staining (**j**)

We characterized 4 of the Cre lines for inducible tissue-specific Cre expression by tamoxifen injection. Brain sections were stained with anti-Cre polyclonal antibody and goat anti-rabbit IgG 2nd antibody conjugated with Alexa fluor 594. Cre expression is detected in Wnt1-Cre (throughout the brain section), Mor23-Cre (hypothalamus and olfactory region), and GAD67-Cre rats (towards to edge of the section associated with mature neurons), but not in Tie2-Cre rat (Fig. 7). Tie2 drives expression in vascular endothelial cells, so serving as a negative control. Nestin-Cre mice (Jax 003771) were used as a positive control. Nestin promotor driven Cre recombinase is expressed in the central and peripheral nervous system, including neuronal and glial cell precursors.

**Fig. 8:**
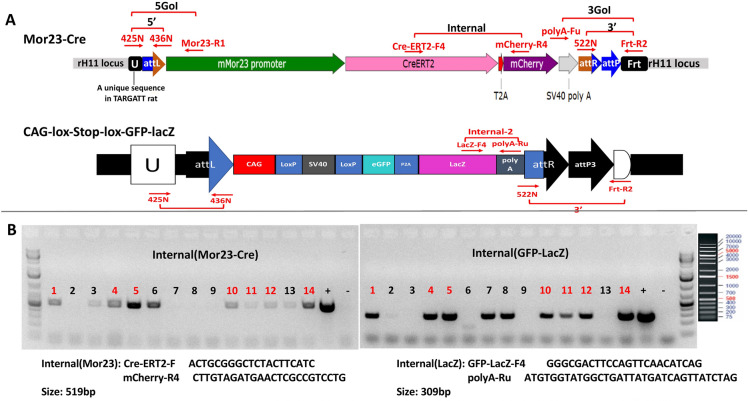
Testing of tissue-specific Cre recombination using reporter line #21 (CAG-lox-Stop-lox-GFP-lacZ). Mor23-Cre/CAG-lox-Stop-lox-GFP-LacZ double transgenic rats were obtained by crossing the two single transgenic line (**a**). Multiple F1 animals containing both transgenes were identified by PCR primers amplifying internal fragments for Mor23-Cre or GFP-lacZ, respectively (**b**). Positive animals are in red No. (**c**) Brain sections of Mor23-Cre/CAG-GFP-lacZ double positive animals were examined for GFP expression by green fluorescence (**b**) or Cre expression (**c**) by antibody staining using anti-Cre antibody

Characterization of Cre-mediated recombination was done by crossing one of the Cre lines, e.g., Mor23-Cre with the tester line#21, CAG-Lox-Stop-Lox-GFP-lacZ, followed by examining GFP expression. Figure 8 shows the scheme for obtaining double transgenic animals containing Mor23-Cre/CAG-GFP-lacZ. Upon Cre recombination, the Stop cassette is deleted and reporter GFP is expressed in the brain mainly in the hypothalamus and olfactory region, coinciding with Cre staining (Fig. 8c).

## Discussion

Lack of appropriate rat models of human diseases is a bottleneck in advancing biomedical research, particularly in neural behavioral and cardiovascular studies. Here we have established a rat model resource containing 10 new Cre rat lines and one Cre test line. These Cre driver lines will enable the creation of physiologically relevant rat models in which gene knockout or expression are regulated in a controlled temporal and spatial manner in vivo. We took a parallel approach using both our proprietary TARGATT technology and CRISPR and were able to establish 11 new rat lines within two years. These lines are now available to researchers through the Rat Resource and Research Center (RRRC).

In comparison of TARGATT generated knockin vs. CRISPR generated knockin models, we found that TARGATT generated lines contain intact transgene insertion, but with some founders containing both site-specific and random insertion. In the case of line#5 (PDGF-CreERT2), only random insertion went germline, whereas line#21 (reporter/test line) had transgenic founders and F1s containing only random insertion events. The knockin efficiency using TARGATT was very low at 1% compared to TARGATT in mice or CRISPR method. This is partly due to the large size of the TARGATT constructs, which is in the range of 8.3 kb to 8.9 kb. Alternatively, the integrase mRNA in the microinjection mixture may be more prone to degradation, affecting its integration activity.

CRISPR generated lines, on the other hand, contain indels or mutations which may affect transgene expression. For example, we initially were able to generate founders for Nestin-CreERT2 (#1) and Plp-CreERT2 (#17), but subsequent breeding and sequencing of the F1 animals showed mutations. While CRISPR was more efficient for generation of transgenic lines, few of these demonstrated good fidelity. For example, in many cases the PCR did not detect 3′ junction and/or 5′ junction fragments. We suspect that large deletions may have happened. Knockin efficiency using CRISPR ranges between 4 and 21%, suggesting that efficiency is locus dependent.

In selecting tissue/cell specific promoters, we gave preferences to those that had previously been proven to work in animal models, as these were most likely to provide predictable regulation in transgenic rats. The human PDGF b-chain promoter used in Line #5 was shown to drive reporter gene expression in the brain, overlapping with antibody expression studies in the non-human primate brain (Sasahara et al. 1991), whereas the human GFAP promoter in Line #16 was shown to direct reporter lacZ expression to astrocytes in transgenic mice and in cultured human cells (Brenner et al. 1994). Line#20 (SMHC-CreERT2) contains the rabbit SMHC promoter shown to direct luciferase expression specifically in vascular smooth muscle cells of large arteries including coronary arteries in transgenic mice and rabbits (Franz et al. 1999). Given these data, these promoters are highly likely to have a similar cell-specific activity in transgenic rats.

Mouse promoters are better characterized and studied compared to rat gene promoters. Line #7 uses the mouse MOR23 promoter to drive Cre expression to olfactory sensory neurons. Characterization of cell-specific expression in this line was confirmed to be in hypothalamus and olfactory region, similar to the expression pattern in transgenic mice (Vassalli et al. 2002).

## Data Availability

The datasets generated during and/or analyzed during the current study are available from the corresponding author upon reasonable request.
