# Correction: Establishment of a Cre-rat resource for creating conditional and physiological relevant models of human diseases

**DOI:** 10.1007/s11248-026-00501-z

**Published:** 2026-06-30

**Authors:** Huimin Zhang, Qi Zheng, Ruby Yanru Chen-Tsai

**Affiliations:** Applied StemCell, Inc, Milpitas, CA USA

**Correction to: Transgenic Res (2021) 30:91–104** **https://doi.org/10.1007/s11248-020-00226-7**

In this article, part C of Figure 8 is missing. The correct Fig. 8 can be found here.

**Fig. 8:**
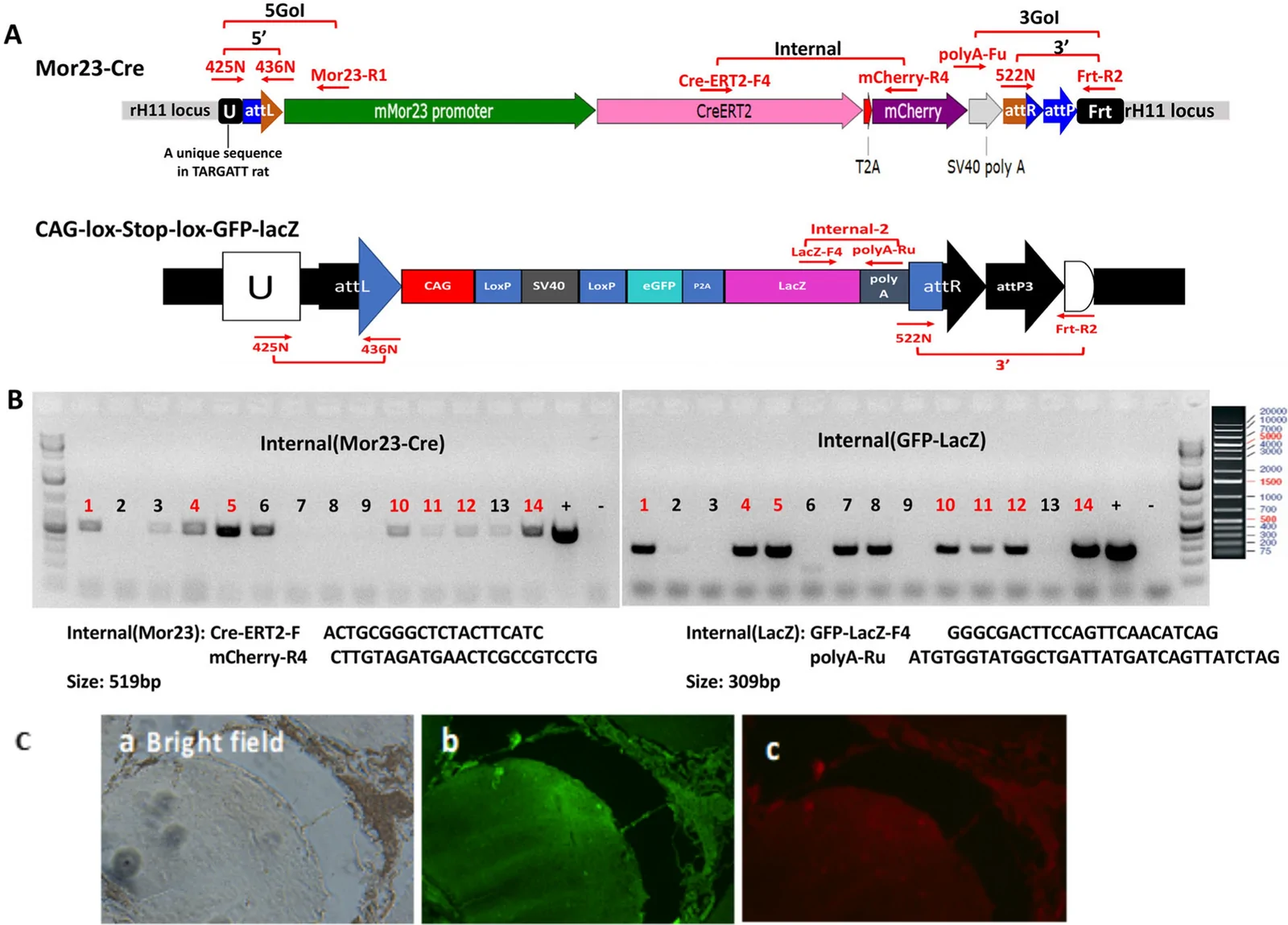
Testing of tissue-specific Cre recombination using reporter line #21 (CAG-lox-Stop-lox-GFP-lacZ). Mor23-Cre/ CAG-lox-Stop-lox-GFP-LacZ double transgenic rats were obtained by crossing the two single transgenic line (**a**). Multiple F1 animals containing both transgenes were identified by PCR primers amplifying internal fragments for Mor23-Cre or GFPlacZ, respectively (**b**). Positive animals are in red No. (**c**) Brain sections of Mor23-Cre/CAG-GFP-lacZ double positive animals were examined for GFP expression by green fluorescence (**b**) or Cre expression (**c**) by antibody staining using anti-Cre antibody

